# Prevalence and Associated Factors of Keratoconus in Latin America and the Caribbean: A Systematic Review and Meta-Analysis

**DOI:** 10.3390/vision10030049

**Published:** 2026-08-01

**Authors:** Kingsley Ekemiri, Chioma Ekemiri, Deo Singh, Robin Seemongal-Dass, Melkamu Temeselew Tegegn, Alicia Hartman, Genalin Ang, Onohomo Adebo, Obinna Awiaka, Obinna Ebere, Oluwasola Michael Ojo, Chigozie John Ekenze

**Affiliations:** 1 Department of Optometry, Faculty of Health Sciences, Abia State University, Uturu P.M.B. 2000, Nigeria; obinna.ebere@abiastateuniversity.edu.ng; 2 Optometry Unit, Department of Clinical Surgical Sciences, Faculty of Medical Sciences, The University of the West Indies, St Augustine Campus, St Augustine City P.O. Box 139, Trinidad and Tobago; 3 School of Nursing, Faculty of Medical Sciences, The University of the West Indies, St Augustine Campus, St Augustine City P.O. Box 139, Trinidad and Tobago; chiomaunakalamba@gmail.com; 4 Department of Ophthalmology, Caribbean Eye Institute, Igneri Road, Valsayn City 0000, Trinidad and Tobago; singh_deo@hotmail.com; 5 Ophthalmology Unit, Department of Clinical Surgical Sciences, Faculty of Medical Sciences, The University of the West Indies, St Augustine Campus, St Augustine City P.O. Box 139, Trinidad and Tobago; robin.seemongal-dass@uwi.edu; 6 Department of Optometry, College of Medicine and Health Sciences, University of Gondar, Gondar P.O. Box 196, Ethiopia; melkamuopta@gmail.com; 7 Department of Optometry, Eye Q Stylist Optician, Bridgetown City BB11000, Barbados; aliciatharts@gmail.com; 8 Department of Optometry, Faculty of Health Sciences, University of Guyana, Georgetown City P.O. Box 101110, Guyana; genalin.ang@uog.edu.gy (G.A.); onohomo.adebo@uog.edu.gy (O.A.); 9 The Office of the Registrar, Optometrists and Dispensing Opticians Registration Board of Nigeria, Abuja City 900101, Nigeria; obinnaawiaka@yahoo.com; 10 Department of Optometry & Vision Science, Faculty of Health Sciences, University of Ilorin, Ilorin City P.M.B. 1515, Nigeria; ojo.om@unilorin.edu.ng; 11 Department of Optometry, Faculty of Health Sciences, Imo State University, Owerri P.M.B. 2000, Nigeria; chigozieekenze2@gmail.com

**Keywords:** keratoconus, epidemiology, prevalence, risk factors, Latin America, Caribbean

## Abstract

Purpose: Keratoconus is a progressive corneal ectatic disorder that causes visual impairment, particularly in adolescents and young adults. Evidence on its epidemiology in Latin America and the Caribbean remains limited. This study aimed to estimate the prevalence of keratoconus and evaluate associated factors, including eye rubbing, sex, family history, and allergy history, in the region. Methods: A systematic search of PubMed, Scopus, PsycINFO, and Google Scholar was conducted on 15 December 2025 for observational studies published up to December 2025. Study selection and data extraction were performed independently by two reviewers, with disagreements resolved by consensus. Study quality was assessed using the Newcastle–Ottawa Scale. Pooled prevalence estimates and odds ratios (ORs) were calculated using a random-effects model with variance-stabilizing transformation. Heterogeneity was assessed using the I^2^ statistic, and publication bias was explored using funnel plots and Egger’s test. Results: Seven prevalence studies were included in the primary meta-analysis following exclusion of three studies from the previous version of the review, with no additional eligible prevalence studies identified in the updated search. One additional nationwide registry-based study from Colombia was identified but excluded from the primary prevalence synthesis because it reported incidence derived from administrative diagnostic coding rather than clinically assessed prevalence. Among the seven prevalence studies, reported prevalence ranged from 0.50% to 2.88%. The pooled prevalence of keratoconus was 2.07% (95% CI: 1.53–2.79), with substantial heterogeneity (I^2^ = 71.9%). Subgroup analyses suggested that prevalence estimates varied by study design, with lower estimates observed in community- and school-based studies and higher estimates in clinic-based studies. No statistically significant associations were identified for eye rubbing, sex, family history, or allergy history; however, these analyses should be interpreted cautiously due to small sample sizes, heterogeneous exposure definitions, and wide confidence intervals. Conclusions: Keratoconus prevalence estimates in Latin America and the Caribbean remain variable and should be interpreted cautiously due to substantial heterogeneity, differences in study design, variability in diagnostic criteria, and differences in case ascertainment. The findings highlight important evidence gaps and support the need for well-designed population-based studies using standardized diagnostic criteria to inform screening strategies, early detection, and eye-care planning in the region.

## 1. Introduction

Keratoconus is a bilateral and asymmetric corneal ectatic disorder characterised by progressive thinning and steepening of the cornea, which leads to irregular astigmatism and reduced visual acuity and primarily affects adolescents and early adults [1,2]. The disease course is often progressive during the early decades of life, although older individuals may also remain affected. Visual impairment may occur despite early diagnosis, particularly where progression continues or access to timely intervention is limited.

Keratoconus has a substantial impact on the quality of life of patients, impairing visual performance and limiting daily activities. In addition to its physical and psychological burden, keratoconus is associated with significant direct and indirect economic costs related to long-term clinical monitoring, contact lens (CL) fitting, corneal cross-linking, and, in advanced cases, corneal transplantation [3,4,5,6,7]. Corneal cross-linking is a procedure intended to halt or slow keratoconus progression by strengthening corneal collagen, thereby reducing the risk of further ectatic change and visual deterioration. From a clinical perspective, the increasing recognition of keratoconus has led to a growing demand for corneal cross-linking services, placing pressure on already limited eye-care resources, particularly in low- and middle-income settings.

The prevalence of reported keratoconus varies widely across regions, reflecting differences in geographic location, diagnostic criteria, and study methodology. More than 23.7 million individuals are estimated to be affected globally. In Europe, prevalence in the general population has been reported as approximately 265 cases per 100,000 people, while substantially higher estimates of up to 4790 per 100,000 have been reported in parts of Asia [8,9,10]. Genetic susceptibility, environmental factors, eye rubbing, and ultraviolet exposure are thought to contribute to these regional differences. In recent years, the wider availability of corneal topography and tomography has further increased the detection of early and subclinical diseases, particularly among younger populations, contributing to rising prevalence estimates [11,12,13,14].

Despite the recognised global burden of keratoconus, evidence from Latin America and the Caribbean remains limited. Most epidemiological and clinical studies have focused on populations in Europe, North America, the Middle East, and Asia, where access to specialised corneal imaging and structured screening programmes is more widespread. Latin America and the Caribbean encompass diverse populations with varying genetic backgrounds, environmental exposures, and healthcare access, creating a unique context in which keratoconus may present differently. In many parts of the region, limited access to specialist eye care increases the risk of late presentation, when therapeutic options are more restricted and visual outcomes are poorer.

The lack of region-specific synthesis hampers informed decision-making by clinicians and policymakers. Uncertainty regarding disease burden complicates planning for screening initiatives, allocation of corneal cross-linking services, and development of paediatric screening strategies aimed at early detection and prevention of disease progression. Therefore, a comprehensive synthesis of available evidence is needed to clarify reported prevalence estimates and associated factors in Latin America and the Caribbean and to support evidence-based strategies tailored to the region’s demographic and healthcare contexts for early diagnosis, prevention, and clinical management.

### Objectives for the Review

1. To estimate the pooled prevalence of keratoconus in Latin America and the Caribbean.
2. To assess associations between keratoconus and selected risk factors, including eye rubbing, sex, family history, and allergy history.

## 2. Materials and Methods

### 2.1. Study Design and Search Strategy

This systematic review and meta-analysis were conducted in accordance with the Preferred Reporting Items for Systematic Reviews and Meta-Analyses (PRISMA) 2020 guidelines [14]. A comprehensive literature search was performed using PubMed, Scopus, PsycINFO, and Google Scholar to identify relevant observational studies reporting keratoconus prevalence or associated factors in Latin America and the Caribbean.

The Cochrane POCC framework (Population, Outcome, Condition, and Context) guided the search strategy and incorporated appropriate Medical Subject Headings (MeSH) terms and free-text keywords combined using Boolean operators (“AND” and “OR”). Search terms included “keratoconus,” “corneal diseases,” “conical cornea,” “prevalence,” “incidence,” “genetics,” “risk factors,” “eye rubbing,” and “atopy,” in combination with “Latin America” or “Caribbean.”

Grey literature was identified through Google Scholar searches and manual screening of reference lists of included studies to minimise publication bias and enhance retrieval of non-indexed sources.

The electronic search strategy was peer reviewed using the PRESS (Peer Review of Electronic Search Strategies) guideline by an independent reviewer [13]. The final search was conducted on 15 December 2025.

The full search strategy is provided in Supplementary File S1. For the purposes of this review, “Latin America” was defined as Spanish- and Portuguese-speaking countries and territories of continental Central and South America and Mexico, and “the Caribbean” was defined as the island nations and territories of the Caribbean basin, consistent with the United Nations geoscheme classification.

### 2.2. Protocol Registration

This systematic review and meta-analysis was retrospectively registered on the Open Science Framework (OSF)on 25 October 2025 (DOI: https://doi.org/10.17605/OSF.IO/ZT8QS). The eligibility criteria, search strategy, and planned analyses (pooled prevalence estimation, subgroup analysis by study design/setting, and evaluation of associated risk factors) were applied consistently with the protocol as registered, and no substantive deviations in study selection or analytic approach occurred prior to registration. As the protocol was not prospectively registered, this should nonetheless be considered when interpreting the findings, since undocumented refinements to the eligibility criteria prior to registration cannot be fully excluded.

### 2.3. Eligibility Criteria

#### 2.3.1. Inclusion Criteria

Studies were included if they met the following criteria:

1. Publication period: Studies published up to December 2025.
2. Study design: Observational studies, including cross-sectional, cohort, and case–control studies.
3. Language: Studies published in any language.
4. Population: Studies conducted among adolescents and adults.
5. Geographic scope: Studies conducted in Latin America and the Caribbean, including both published and unpublished sources.

Outcomes: Data on keratoconus prevalence and/or associated risk factors were reported. 

#### 2.3.2. Exclusion Criteria

1. Review articles, systematic reviews, meta-analyses, editorials, commentaries, and case reports.
2. Studies that did not report relevant outcome data on the prevalence of keratoconus or associated factors.

### 2.4. Age Definitions

Adolescents were defined as individuals aged 10–19 years, and adults as individuals aged 20 years and older. No lower age cutoff was imposed in the review; however, the inclusion of younger age groups who may be at risk but not yet clinically diagnosed may have influenced prevalence estimates.

### 2.5. Conceptual Framework

This review did not adopt a Global Burden of Disease framework; instead, it focused on synthesising reported prevalence estimates and associated factors derived from eligible observational studies.

### 2.6. Study Selection Process

Two reviewers (KE and OA) independently screened titles and abstracts, followed by full-text assessment for eligibility. Discrepancies were resolved through discussion and, where necessary, consultation with a third reviewer (CE). Study selection and data extraction were conducted independently using predefined eligibility criteria and a standardised data extraction form. This approach was implemented to minimise selection bias and ensure consistency in study inclusion and data capture across heterogeneous study designs.

### 2.7. Diagnostic Criteria

Studies were included regardless of the diagnostic method used. Diagnostic approaches varied across studies and included clinical examination, corneal topography, corneal tomography, and, in some cases, administrative or registry-based diagnostic coding. The absence of standardised diagnostic criteria across studies may have influenced case identification, particularly for early or subclinical keratoconus, and is considered an important source of heterogeneity in the analysis.

### 2.8. Data Extraction

Following study selection, data extraction was independently performed in pairs using a standardised data extraction form. Extracted data were recorded in a Microsoft Excel spreadsheet and included the following information: first author, year of publication, study design, study setting, sample size, population characteristics, reported prevalence of keratoconus, and data on associated factors, including corresponding odds ratios (ORs) where available.

### 2.9. Data Items and Outcomes

For the analysis of associated factors, variables were extracted when they were reported in two or more studies to allow for meaningful quantitative synthesis. Discrepancies in data extraction were resolved through discussion and consensus among the reviewers. The primary outcome of this review was the prevalence of keratoconus. Secondary outcomes included associations between keratoconus and selected risk factors, expressed as odds ratios.

### 2.10. Study Risk of Bias Assessment

The methodological quality of the included studies was assessed using the Newcastle–Ottawa Scale (NOS), adapted for cross-sectional studies [15]. The scale evaluates study quality across three domains: selection, comparability, and outcome assessment, with a maximum possible score of 10 points.

Studies achieving a NOS score of five or higher were of acceptable methodological quality and were included in the quantitative synthesis [15]. Multiple reviewers independently conducted the quality assessment, and any discrepancies were resolved through discussion and consensus among all authors.

The NOS was selected because it is widely used and readily adaptable across the range of study designs included in this review (community-based, school-based, and clinic-based cross-sectional studies), allowing a single consistent instrument to be applied throughout. We acknowledge that instruments developed specifically for prevalence studies, such as the Joanna Briggs Institute (JBI) Critical Appraisal Checklist for Studies Reporting Prevalence Data, are increasingly recommended for this study type, as they more directly address sampling frame adequacy, response rate, and case definition validity. The adapted NOS was retained here for consistency with prior versions of this review and comparability with related literature; however, future updates of this synthesis should consider dual assessment using the JBI checklist to better capture prevalence-specific sources of bias.

### 2.11. Effect Measures

Proportions were used as the effect measure for keratoconus prevalence, whereas odds ratios (ORs) were used to quantify associations between keratoconus and selected risk factors.

### 2.12. Methods of Data Analysis and Synthesis

Following data extraction, all analyses were conducted using STATA software (version 14). Pooled prevalence estimates and associations were calculated using a random-effects model, selected a priori to account for expected clinical and methodological heterogeneity across studies, including differences in study setting, population characteristics, and diagnostic approaches. A Freeman–Tukey double arcsine transformation was applied to stabilise variance before pooling prevalence estimates, particularly for studies reporting proportions close to 0 or 1 [16].

Between-study heterogeneity was assessed using Cochran’s Q test, the I^2^ statistic was used to quantify the proportion of total variability attributable to heterogeneity, and τ^2^ (tau-squared) was used to estimate between-study variance. Heterogeneity was classified as low (I^2^ < 50%), moderate (50–75%), or high (>75%) [17]. A *p* value < 0.05 for Cochran’s Q or τ^2^ was considered indicative of statistically significant heterogeneity beyond chance.

A Freeman–Tukey double arcsine transformation was applied to stabilise variance prior to pooling prevalence estimates, particularly given that several included studies reported proportions close to the lower bound (0.50–2.88%). This transformation was selected because it is well established for prevalence meta-analyses in ophthalmology and reduces distortion when raw proportions are combined under a standard random-effects model. We recognise that this approach has been criticised in recent methodological literature, principally because back-transformation can occasionally produce implausible estimates and because it does not explicitly model the binomial nature of the underlying data. Generalised linear mixed models (GLMMs) using a logit or complementary log-log link have been proposed as preferable alternatives, as they model within-study variance more directly and avoid back-transformation artefacts. Given the modest number of studies (n = 7) and the presence of studies with small sample sizes, we retained the Freeman–Tukey approach for consistency with the broader keratoconus prevalence literature; nonetheless, a GLMM-based sensitivity analysis is recommended for future iterations of this review to confirm the robustness of the pooled estimate to choice of transformation.

To explore potential sources of heterogeneity, subgroup analyses were performed according to geographic region (Latin America versus the Caribbean). Where data permitted, subgroup analyses were also conducted according to study setting, study design, and data source to further examine methodological sources of heterogeneity. Publication bias was explored through visual inspection of funnel plots and Egger’s regression test; however, these approaches were interpreted cautiously because of the small number of included studies and the known limitations of publication-bias assessment in prevalence meta-analyses. Studies published in languages other than English were translated where necessary. To examine the robustness of pooled estimates, sensitivity analyses were conducted using a leave-one-out approach, whereby individual studies were sequentially excluded.

Between-study variance was additionally quantified using τ^2^ alongside the I^2^ statistic, and 95% prediction intervals were calculated for the pooled prevalence estimate to indicate the range within which the true prevalence would be expected to fall in a new, comparable setting. A formal test for subgroup differences (Q-test between subgroups) was conducted to determine whether pooled prevalence differed significantly between community-/school-based and clinic-based studies.

### 2.13. Certainty of Evidence

This review did not incorporate a formal assessment of certainty of evidence using frameworks such as GRADE. This represents a limitation, as such an assessment would allow readers to gauge confidence in the pooled prevalence and risk-factor estimates beyond point estimates and heterogeneity statistics alone. Given the substantial heterogeneity, small number of studies for several risk-factor analyses, and reliance on non-standardised diagnostic criteria, the overall certainty of evidence for both the pooled prevalence estimate and the risk-factor associations is judged to be low, and formal GRADE assessment is recommended in future updates of this review.

## 3. Results

### 3.1. Selection and Characteristics of Studies

**Figure 1:**
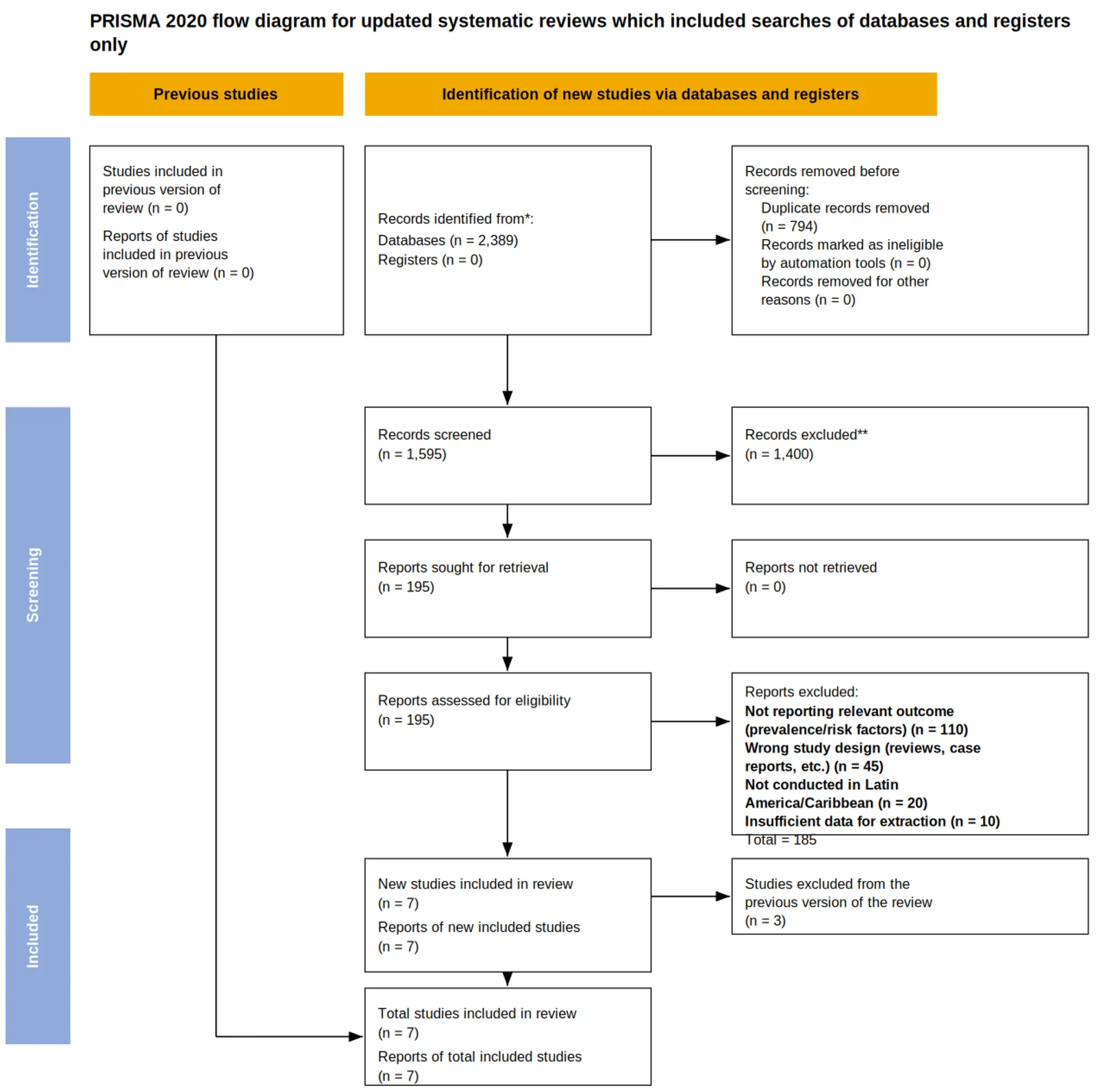
PRISMA flowchart diagram of the study selection process. * Consider, if feasible to do so, reporting the number of records identified from each database or register searched (rather than the total number across all databases/registers). ** If automation tools were used, indicate how many records were excluded by a human and how many were excluded by automation tools.

The literature search was conducted on 15 December 2025 and identified 2389 records, including 1627 from PubMed, 451 from Scopus, 299 from PsycINFO, and 12 from Google Scholar. After duplicate removal, 1595 records remained for screening. Following title and abstract screening, 195 articles were assessed in full for eligibility, of which 185 were excluded for not meeting the inclusion criteria. Seven prevalence studies were included in the primary meta-analysis following exclusion of three studies from the previous version of the review, with no additional eligible prevalence studies identified in the updated search (Figure 1).

The seven studies included in the primary prevalence meta-analysis varied in design and setting, comprising community-based, school-based, and clinic-based cross-sectional studies. Most studies were conducted in Latin America, with limited representation from the Caribbean. Individual sample sizes among the prevalence studies ranged from 274 to 91,426 participants, with a combined sample size of 95,955. The characteristics of the seven prevalence studies included in the primary meta-analysis are presented in Table 1.

Table 2 summarises the diagnostic approach used in each of the seven included prevalence studies, illustrating the variability in case ascertainment that likely contributed to the observed heterogeneity. The diagnostic approach varied considerably across studies, ranging from self-reported or questionnaire-based ascertainment to clinical examination and instrument-based corneal topography or tomography. This variability in case ascertainment represents a key source of between-study heterogeneity, as studies relying on self-report or non-instrumented clinical examination may under-detect subclinical or early-stage disease relative to studies using corneal tomography.

One additional nationwide registry-based study from Colombia was identified. This study included 50,372,424 individuals and reported keratoconus incidence using administrative diagnostic coding rather than clinically assessed prevalence. Because incidence and prevalence represent different epidemiological measures, this study was excluded from the primary prevalence meta-analysis and retained only for descriptive and contextual interpretation. The nationwide Colombian registry-based incidence study is presented separately in Table 3.

Pooled prevalence estimates were calculated using the DerSimonian and Laird random-effects model. Registry-based studies reporting incidence or administrative data were not included in the primary prevalence meta-analysis. Statistical heterogeneity was assessed using the I^2^ statistic.

In the primary meta-analysis restricted to studies reporting prevalence, the pooled prevalence of keratoconus was 2.07% (95% CI: 1.53–2.79), with substantial heterogeneity (I^2^ = 71.9%; τ^2^ = 0.24; 95% prediction interval: 0.4–3.3%). Subgroup analyses demonstrated that study design influenced prevalence estimates. When restricted to community- and school-based studies, the pooled prevalence was 1.34% (95% CI: 0.60–2.95), whereas clinic-based studies yielded higher estimates (2.84%; 95% CI: 2.73–2.94), likely reflecting selection bias and differences in case ascertainment.

A formal test for subgroup differences was conducted to determine whether the observed difference in pooled prevalence between community-/school-based studies (1.34%; 95% CI: 0.60–2.95) and clinic-based studies (2.84%; 95% CI: 2.73–2.94) was statistically significant (Q_between = 6.21, df = 1, *p* = 0.013). This difference was statistically significant, confirming that pooled prevalence differed meaningfully according to study setting. This finding supports study setting as a genuine, rather than incidental, contributor to the observed heterogeneity, consistent with selection bias and differential case ascertainment across community, school, and clinic populations.

**Figure 2:**
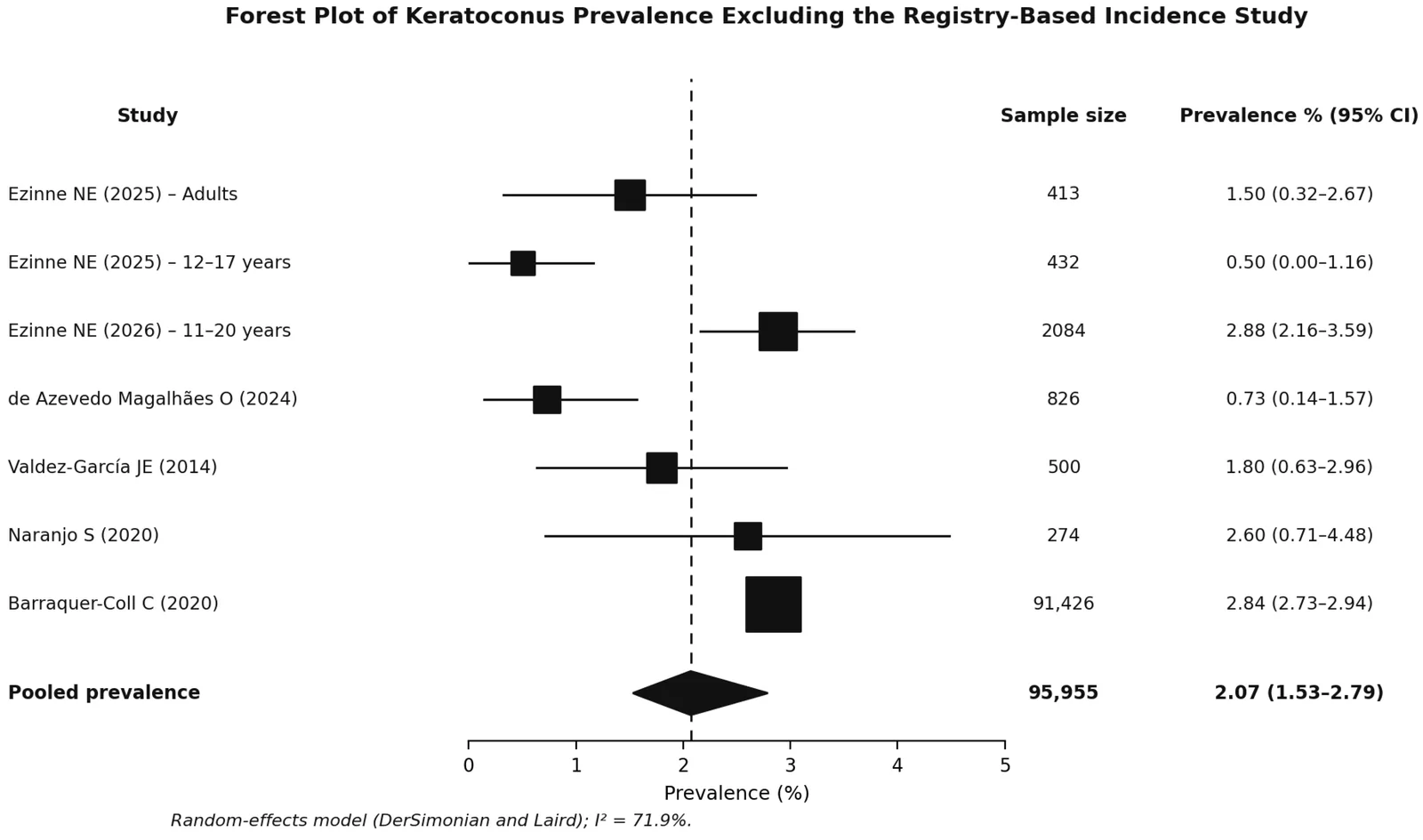
Forest plot of keratoconus prevalence based on studies included in the primary prevalence meta-analysis [18,19,20,21,22,23,24]. Each square represents the point prevalence estimate of an individual study, and the size of each square is proportional to the study’s weight in the meta-analysis (i.e., to its sample size and inverse variance). The horizontal line through each square indicates the 95% confidence interval (CI) of that estimate. The diamond represents the pooled prevalence estimate, with its centre indicating the pooled value and its width representing the corresponding 95% CI. The vertical dashed line marks the pooled prevalence estimate. Pooled prevalence was calculated using a random-effects model (DerSimonian and Laird); between-study heterogeneity was I^2^ = 71.9%. The registry-based incidence study was excluded from this analysis because it reported incidence rather than clinically assessed prevalence

These findings indicate that study design, sampling frame, and diagnostic approach substantially influenced reported prevalence estimates. The forest plot of keratoconus prevalence based on studies included in the primary prevalence meta-analysis is presented in Figure 2.

### 3.2. Heterogeneity and Publication Bias

Substantial heterogeneity was observed across studies included in the prevalence meta-analysis (I^2^ = 71.9%), reflecting important methodological differences in study design, population characteristics, and diagnostic approaches. Previous sensitivity analyses indicated that a high-prevalence study substantially influenced pooled estimates; however, after restricting the primary synthesis to eligible prevalence studies, heterogeneity remained substantial, suggesting that differences in study design, diagnostic criteria, sampling frame, and age distribution continued to contribute to between-study variability. Differences in age distribution across studies may also have contributed to heterogeneity, as keratoconus is more prevalent in adolescents and young adults. Studies including broader age ranges or adult-only populations may therefore underestimate or overestimate prevalence, depending on the age structure of the sampled population. Given this anticipated heterogeneity, subgroup analyses were conducted based on study setting and data source. These analyses demonstrated that prevalence estimates varied systematically by study design, with lower estimates observed in community- and school-based studies and higher estimates in clinic-based studies, suggesting the influence of selection bias and differences in case ascertainment.

Meta-regression analyses were performed to explore potential sources of heterogeneity using sample size and publication year as covariates. Neither variable was significantly associated with between-study heterogeneity (*p* = 0.052 and *p* = 0.411, respectively), indicating that these factors did not account for the observed variability.

**Figure 3:**
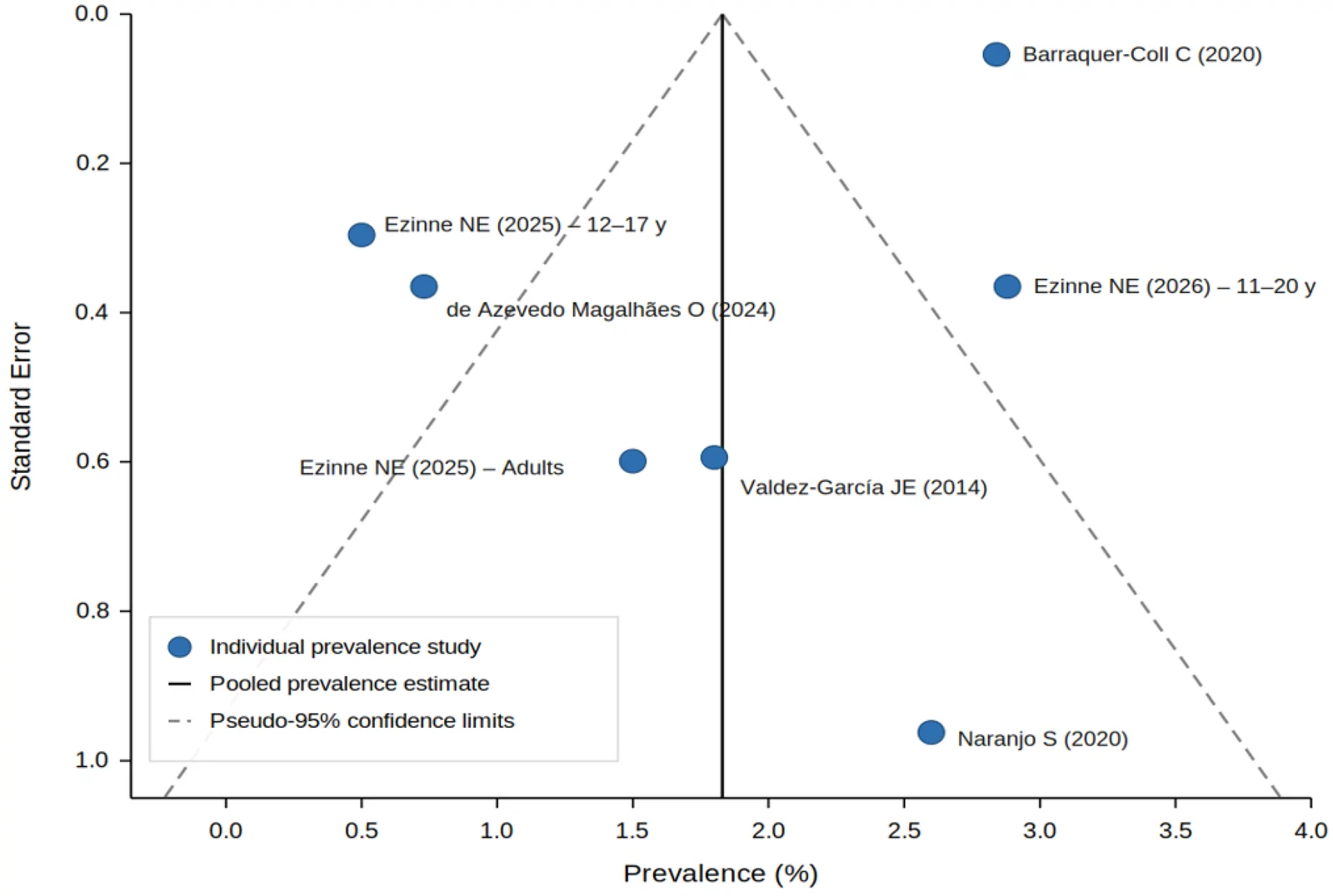
Funnel plot assessing publication bias after exclusion of the dominant registry-based study. Each dot represents an individual included prevalence study, plotted as its prevalence estimate (*x*-axis) against its standard error (*y*-axis). The vertical line indicates the pooled prevalence estimate, and the two diagonal lines together denote the pseudo-95% confidence limits of the funnel; the colours distinguish only the left and right boundaries and carry no other meaning. In the absence of publication bias, studies are expected to fall symmetrically within the funnel. Interpretation is limited by the small number of studies [18,19,20,21,22,23,24].

Publication bias was assessed using Egger’s regression test and visual inspection of a funnel plot. Egger’s test did not demonstrate statistically significant evidence of publication bias (*p* = 0.086). However, visual inspection of the funnel plot suggested some asymmetry, which is more likely attributable to between-study heterogeneity and differences in study design rather than publication bias alone. A funnel plot excluding the dominant registry-based study is shown in Figure 3.

### 3.3. Sensitivity Analysis

A leave-one-out sensitivity analysis was conducted to evaluate the influence of individual studies on the pooled prevalence estimate. In a secondary sensitivity analysis that included the registry-based study, exclusion of this study resulted in substantially wider confidence intervals, confirming that its exceptionally large sample size influenced the precision of the pooled estimate. However, because the study reported incidence derived from administrative diagnostic coding rather than clinically assessed prevalence, it was excluded from the primary prevalence meta-analysis.

### 3.4. Risk Factors Associated with Keratoconus

Four factors were evaluated for potential associations with keratoconus based on the available data. Pooled analysis of eye rubbing, derived from four studies including 3467 participants, did not demonstrate a statistically significant association with keratoconus (odds ratio [OR] = 0.62; 95% CI: 0.02–21.6; *p* = 0.832). Substantial heterogeneity was observed (I^2^ = 97.3%, *p* < 0.001), likely reflecting differences in exposure definitions, reliance on self-reported behaviour, recall bias, and low event counts.

Three studies comprising 2931 participants assessed the association between sex and keratoconus. No statistically significant association was identified (OR = 0.66; 95% CI: 0.41–1.08; *p* = 0.101), with no detectable heterogeneity (I^2^ = 0.0%, *p* = 0.433), indicating consistent findings across studies.

Analysis of family history from four studies (n = 3402) showed no statistically significant association with keratoconus (OR = 1.27; 95% CI: 0.02–57.2; *p* = 0.900), although this estimate was characterised by considerable heterogeneity (I^2^ = 98.2%, *p* < 0.001) and wide confidence intervals, reflecting substantial uncertainty.

Three studies including 1313 participants evaluated allergy history and found no statistically significant association with keratoconus (OR = 0.70; 95% CI: 0.25–1.94; *p* = 0.495), with low to moderate heterogeneity (I^2^ = 40.2%, *p* = 0.188) (Table 3).

Although no statistically significant associations were observed between keratoconus and the evaluated factors, these findings should be interpreted cautiously. The lack of statistical significance may reflect methodological limitations rather than true absence of association. Given the wide confidence intervals and very high heterogeneity observed for eye rubbing (I^2^ = 97.3%) and family history (I^2^ = 98.2%), a pooled odds ratio may convey a false sense of a single unified effect where none can credibly be assumed to exist. A narrative synthesis, describing the direction and magnitude of association reported in each individual study without collapsing them into a single summary statistic, may therefore provide a more transparent and less potentially misleading representation of the current evidence for these two factors. We report the pooled estimates for completeness and comparability with prior reviews, but recommend that readers weight the narrative pattern across studies, rather than the pooled point estimate, when interpreting these two associations. Sex and allergy history, in contrast, showed low heterogeneity (I^2^ = 0.0% and 40.2%, respectively) and are more appropriately summarized by the pooled estimate. The analyses were constrained by small sample sizes, heterogeneous exposure definitions, reliance on self-reported data, limited adjustment for confounding variables, and wide confidence intervals, which reduced the ability to detect meaningful associations (Table 4).

## 4. Discussion

The progression of keratoconus is most pronounced during the early years following disease onset, with corneal steepening typically occurring within the first two decades of life [2]. Early diagnosis remains challenging because initial structural changes may be subtle and difficult to detect using conventional clinical examination alone [12]. Although keratoconus has traditionally been viewed as a condition that stabilises with age, longitudinal studies indicate that corneal topographic changes may continue beyond the third decade, even in non-contact lens wearers [2]. Posterior corneal steepening and thinning have also been reported after the age of 40 years, although these changes are generally modest and of limited clinical significance [1].

A key finding of this review is that reported prevalence estimates are highly dependent on study design. Community- and school-based studies are more likely to approximate true population prevalence, whereas clinic-based studies are subject to selection bias and may overestimate disease frequency due to enrichment of symptomatic or advanced cases. Conversely, registry-based studies relying on administrative diagnostic codes may underestimate prevalence, as they capture only diagnosed cases within healthcare systems and are influenced by access to care and coding practices. These methodological differences likely explain the substantial heterogeneity observed across studies. Differences in age distribution across studies may also have contributed to heterogeneity, as keratoconus is more prevalent in adolescents and young adults. Consequently, studies including broader age ranges or adult-only populations may underestimate or overestimate prevalence depending on the age structure of the sampled population.

Beyond study design, diagnostic modality itself is likely to have shaped case ascertainment independently of setting. Studies relying on clinical examination alone may under-detect early or subclinical keratoconus, whereas studies incorporating corneal topography or tomography are more likely to identify forme fruste or early-stage diseases that would otherwise go unrecognized [12]. This distinction is conceptually separate from the community/clinic-based dichotomy discussed above: a clinic-based study using only slit-lamp examination and a community-based study using tomographic screening could, in principle, yield divergent prevalence estimates for reasons unrelated to sampling frame. The seven included studies varied in diagnostic approach in this way, and this variability is considered an additional, only partially overlapping, contributor to the substantial heterogeneity (I^2^ = 71.9%) observed in the primary meta-analysis.

The predominance of clinic-based studies in this review may have influenced not only the pooled prevalence estimate but also the observed distribution of associated risk factors. Patients presenting to specialist eye-care clinics are disproportionately likely to be symptomatic for example, reporting eye rubbing or a family history of keratoconus, which may distort the apparent strength or direction of these associations relative to a true population-level relationship. This selection mechanism, sometimes termed collider or ascertainment bias, could plausibly contribute to the instability of the eye-rubbing and family-history estimates described above, independent of exposure misclassification or recall bias.

In this study, the primary pooled prevalence of keratoconus, based on studies specifically reporting prevalence, was 2.07% (95% CI: 1.53–2.79). Inclusion of the large registry-based study increased the pooled estimate to 5.35% (95% CI: 3.80–6.91), highlighting its disproportionate influence on the meta-analysis due to its exceptionally large sample size. Therefore, the 2.07% estimate is considered the more clinically meaningful representation of keratoconus prevalence in Latin America and the Caribbean. This estimate appears to be higher than previously reported global figures (approximately 0.14%) [23]; however, this discrepancy should be interpreted cautiously. Differences in study design, age distribution, diagnostic criteria, and reliance on clinic-based samples, as well as regional genetic and environmental factors, are likely to have contributed to higher prevalence estimates in this review.

Subgroup analyses demonstrated substantial regional variation, with higher pooled prevalence estimates observed in Latin America than in the Caribbean. These differences are more likely to reflect variability in population characteristics, access to specialist eye care, availability of corneal imaging technologies, and screening practices than true biological variation alone. The limited number of studies from Caribbean settings highlights an important evidence gap and underscores the need for population-based data from this region. Addressing this gap will likely require targeted collaboration between regional academic centres and local eye-care networks for example, integrating keratoconus case identification into existing school vision-screening programmes or optometric referral networks in Caribbean territories rather than relying on opportunistic inclusion of clinic-based data. Establishing a standing regional surveillance mechanism, potentially modelled on the registry-based approach used in Colombia [24], could over time generate more representative, population-based prevalence data for the Caribbean specifically.

This meta-analysis did not identify statistically significant population-level associations between keratoconus and commonly cited factors, including eye rubbing, sex, family history, or allergy history. These findings should not be interpreted as refuting established biological mechanisms linking these exposures to keratoconus. This caution is particularly warranted for eye rubbing and family history, where heterogeneity was extremely high (I^2^ = 97.3% and 98.2%, respectively) and confidence intervals were correspondingly wide (e.g., family history: OR 1.27, 95% CI 0.02–57.2). Estimates of this magnitude of imprecision indicate that the pooled odds ratios for these two factors are exploratory rather than definitive, and should not be used to inform clinical or public health conclusions about the presence or absence of an association. Rather, the null findings across all four factors likely reflect limitations of the available evidence, including heterogeneous exposure definitions, reliance on self-reported data, limited adjustment for confounding, and small event counts, rather than true absence of association. The associations between eye rubbing and keratoconus reported in some clinic-based studies may additionally be influenced by recall bias and reverse causality. Keratoconus is increasingly recognised as a multifactorial condition arising from complex interactions between genetic susceptibility and environmental exposure. Therefore, single-factor analyses may be insufficient to capture disease aetiology at the population level [25,26]. To clarify risk pathways and disease progression, well-designed longitudinal studies incorporating standardised diagnostic criteria and genetically informed approaches are needed.

Prevalence, as estimated in this review, refers to the proportion of a population found to have keratoconus at a given point in time; it should be distinguished from the broader concept of disease burden, which additionally incorporates disease severity, progression, visual disability, and downstream economic and quality-of-life impacts. The present synthesis speaks only to the former; conclusions about regional disease burden would require complementary data on severity distribution, complication rates, and healthcare utilisation, which were beyond the scope of the included studies.

These findings highlight the potential under-recognition of keratoconus in Latin America and the Caribbean, particularly among younger populations, where earlier detection may help prevent disease progression and reduce long-term visual morbidity. At a population level, earlier identification of keratoconus also has important implications for contact lens management, as timely diagnosis may delay progression to rigid contact lens dependence and help preserve long-term visual outcomes. Improved access to screening, adoption of standardised diagnostic protocols, and population-based surveillance are essential to inform service planning, optimise timely intervention, and guide preventive strategies across the region.

## 5. Conclusions

This systematic review and meta-analysis demonstrates that reported prevalence estimates of keratoconus in Latin America and the Caribbean are highly variable and appear higher than those reported in some global estimates, suggesting that the regional disease burden may be under-recognised. However, these findings should be interpreted with caution due to substantial methodological heterogeneity and the predominance of clinic-based studies, which may overestimate disease frequency.

Although keratoconus is widely recognised as a multifactorial condition involving complex genetic and environmental interactions, no consistent population-level associations were identified for commonly reported risk factors, including eye rubbing, sex, family history, and allergy history. These findings likely reflect limitations in the available evidence rather than the absence of true associations.

Overall, the wide variability in prevalence estimates underscores significant evidence gaps in the region. Future research should prioritise well-designed population-based studies using standardised diagnostic criteria, alongside the development of regional surveillance systems and targeted screening strategies to facilitate earlier detection and inform effective eye-care planning.

## 6. Limitations of the Study

Additional limitations should be acknowledged. First, substantial variability in diagnostic criteria (e.g., clinical examination, corneal topography, or corneal tomography) likely contributed to the high between-study heterogeneity. Second, the inclusion of a very large registry-based dataset may have disproportionately influenced pooled prevalence estimates. The influence of the large registry-based study was further reflected in sensitivity analyses, where its exclusion resulted in reduced precision of pooled estimates. Third, the assessment of publication bias is limited by the small number of included studies, thereby reducing the reliability of funnel plots and Egger’s regression test. A major limitation of this review is the inclusion of studies with fundamentally different designs, including population-based, clinic-based, and registry-based data sources, which are not directly comparable and may introduce bias into pooled estimates.

Fourth, the review has limited representation in Caribbean settings, which constrains the generalizability of the pooled and subgroup estimates to that subregion specifically; targeted regional surveillance and collaboration with local eye-care networks, as discussed above, may help address this evidence gap in future iterations of the review.

Finally, the absence of comparable SRs or population-based studies from similar settings constrained the contextual comparison of findings.

## Figures and Tables

**Table 1:** Characteristics of studies included in the primary prevalence meta-analysis.

Author (Year)	Country	Age Group	Study Design	Sample Size	Prevalence % (95% CI)
Ezinne NE (2025) [18]	Trinidad and Tobago	≥20 years	Community-based cross-sectional	413	1.50 (0.32–2.67)
Ezinne NE (2025) [19]	Trinidad and Tobago	12–17 years	School-based cross-sectional	432	0.50 (0.00–1.16)
Ezinne NE (2026) [20]	Trinidad and Tobago	11–20 years	School-based cross-sectional	2084	2.88 (2.16–3.59)
de Azevedo Magalhães O (2024) [21]	Brazil	14–21 years	School-based cross-sectional	826	0.73 (0.14–1.57)
Valdez-García JE (2014) [22]	Mexico	10–20 years	Clinic-based retrospective cross-sectional	500	1.80 (0.63–2.96)
Naranjo S (2023) [23]	Colombia	≥18 years	Clinic-based retrospective cross-sectional	274	2.60 (0.71–4.48)
Barraquer-Coll C (2020) [24]	Colombia	All ages	Clinic-based retrospective cross-sectional	91,426	2.84 (2.73–2.94)
**Total**	—	—	—	95,955	

*CI = confidence interval.*

**Table 2:** Diagnostic criteria used across studies included in the primary prevalence meta-analysis.

Author (Year)	Country	Study Design	Diagnostic Method	Diagnostic Basis
Ezinne NE (2025) [18]—Adults	Trinidad and Tobago	Community-based cross-sectional	Self-reported diagnosis/questionnaire-based ascertainment *	Non-instrumented
Ezinne NE (2025) [19]—12–17 years	Trinidad and Tobago	School-based cross-sectional	Clinical examination/optometric screening *	Clinical (non-tomographic)
Ezinne NE (2026) [20]—11–20 years	Trinidad and Tobago	School-based cross-sectional	Clinical examination/optometric screening *	Clinical (non-tomographic)
de Azevedo Magalhães O (2024) [21]	Brazil	School-based cross-sectional	Corneal topography *	Instrument-based
Valdez-García JE (2014) [22]	Mexico	Clinic-based retrospective cross-sectional	Corneal topography/tomography (clinical records) *	Instrument-based
Naranjo S (2023) [23]	Colombia	Clinic-based retrospective cross-sectional	Clinical diagnosis (chart review) *	Clinical/instrument-based
Barraquer-Coll C (2020) [24]	Colombia	Clinic-based retrospective cross-sectional	Corneal topography/tomography (clinical records) *	Instrument-based
Mejia-Salgado G (2023) [25]—excluded from primary synthesis	Colombia	Registry/database-based	Administrative diagnostic coding (ICD-based) *	Non-clinical/administrative

* Diagnostic method as reconstructed from the original publications; every entry must be verified directly against the source paper before finalising this table, including finer detail such as Placido-disc vs. *Scheimpflug tomography or specific staging criteria (*e.g.*, Amsler–Krumeich) where reported.*

**Table 3:** Registry-based keratoconus incidence study identified in the review but excluded from the primary prevalence meta-analysis.

Author (Year)	Country	Age Group	Study Design	Sample Size	Epidemiological Measure
Mejia-Salgado G (2023) [25]	Colombia	All ages	Registry/ database-based retrospective study	50,372,424	Incidence: 10.36 per 100,000 inhabitants (95% CI 10.08–10.64)

*This study was excluded from the primary prevalence meta-analysis because it reported incidence derived from administrative diagnostic coding rather than clinically assessed prevalence. The study is presented separately for contextual interpretation and discussion of keratoconus epidemiology in Latin America.*

**Table 4:** Factors associated with keratoconus in Latin America and the Caribbean.

Determinant	Comparison	No. of Studies	Sample Size	Odds Ratio (95% CI)	*p*-Value	I^2^ (%)	Heterogeneity (*p*-Value)
Eye rubbing	Yes vs. No	4	3467	0.62 (0.02–21.6)	0.832	97.3	<0.001
Sex	Male vs. Female	3	2931	0.66 (0.41–1.08)	0.101	0.0	0.433
Family history	Yes vs. No	4	3402	1.27 (0.02–57.2)	0.900	98.2	<0.001
Allergy history	Yes vs. No	3	1313	0.70 (0.25–1.94)	0.495	40.2	0.188

CI = confidence interval; OR = odds ratio. Estimates should be interpreted with caution due to heterogeneity, small sample sizes, and variability in exposure definitions across studies.

## Data Availability

All data analysed during the current systematic review and meta-analysis are available in the Supporting Information files.

## Supplementary Materials

The following supporting information can be downloaded at: https://www.mdpi.com/article/10.3390/vision10030049/s1, Supplementary File S1, PRISMA 2020 checklist.
